# Patterns of Suicide Ideation Across Eight Countries in Four Continents During the COVID-19 Pandemic Era: Repeated Cross-sectional Study

**DOI:** 10.2196/32140

**Published:** 2022-01-17

**Authors:** Philip J Schluter, Mélissa Généreux, Kevin KC Hung, Elsa Landaverde, Ronald P Law, Catherine Pui Yin Mok, Virginia Murray, Tracey O'Sullivan, Zeeshan Qadar, Mathieu Roy

**Affiliations:** 1 School of Health Sciences University of Canterbury - Te Whare Wananga o Waitaha Christchurch New Zealand; 2 Primary Care Clinical Unit School of Clinical Medicine The University of Queensland Brisbane Australia; 3 Department of Community Health Sciences Faculté de Médecine et des Sciences de la Santé Université de Sherbrooke Sherbrooke, QC Canada; 4 Accident and Emergency Medicine Academic Unit Faculty of Medicine The Chinese University of Hong Kong Hong Kong China; 5 Department of Health Manila Philippines; 6 The Jockey Club School of Public Health and Primary Care Faculty of Medicine The Chinese University of Hong Kong Hong Kong China; 7 United Kingdom Health Security Agency London United Kingdom; 8 Interdisciplinary School of Health Sciences Faculty of Health Sciences University of Ottawa Ottawa, ON Canada; 9 National Collaborating Centre for Infectious Diseases Rady Faculty of Health Sciences University of Manitoba Winnipeg, MB Canada; 10 Department of Family Medicine & Emergency Medicine Faculté de Médecine et des Sciences de la Santé Université de Sherbrooke Sherbrooke, QC Canada

**Keywords:** pandemic, infodemic, psychosocial impacts, sense of coherence, suicide ideation, epidemiology, suicide, pattern, COVID-19, cross-sectional, mental health, misinformation, risk, prevalence, gender, age, sociodemographic

## Abstract

**Background:**

The COVID-19 pandemic and countries’ response measures have had a globally significant mental health impact. This mental health burden has also been fueled by an infodemic: an information overload that includes misinformation and disinformation. Suicide, the worst mental health outcome, is a serious public health problem that can be prevented with timely, evidence-based, and often low-cost interventions. Suicide ideation, one important risk factor for suicide, is thus important to measure and monitor, as are the factors that may impact on it.

**Objective:**

This investigation had 2 primary aims: (1) to estimate and compare country-specific prevalence of suicide ideation at 2 different time points, overall and by gender and age groups, and (2) to investigate the influence of sociodemographic and infodemic variables on suicide ideation.

**Methods:**

A repeated, online, 8-country (Canada, the United States, England, Switzerland, Belgium, Hong Kong, Philippines, and New Zealand), cross-sectional study was undertaken with adults aged ≥18 years, with measurement wave 1 conducted from May 29, 2020 to June 12, 2020 and measurement wave 2 conducted November 6-18, 2021. Self-reported suicide ideation was derived from item 9 of the Patient Health Questionnaire-9 (PHQ-9). Age-standardized suicide ideation rates were reported, a binomial regression model was used to estimate suicide ideation indication rates for each country and measurement wave, and logistic regression models were then employed to relate sociodemographic, pandemic, and infodemic variables to suicide ideation.

**Results:**

The final sample totaled 17,833 adults: 8806 (49.4%) from measurement wave 1 and 9027 (50.6%) from wave 2. Overall, 24.2% (2131/8806) and 27.5% (2486/9027) of participants reported suicide ideation at measurement waves 1 and 2, respectively, a difference that was significant (*P*<.001). Considerable variability was observed in suicide ideation age-standardized rates between countries, ranging from 15.6% in Belgium (wave 1) to 42.9% in Hong Kong (wave 2). Frequent social media usage was associated with increased suicide ideation at wave 2 (adjusted odds ratio [AOR] 1.47, 95% CI 1.25-1.72; *P*<.001) but not wave 1 (AOR 1.11, 95% CI 0.96-1.23; *P*=.16). However, having a weaker sense of coherence (SOC; AOR 3.80, 95% CI 3.18-4.55 at wave 1 and AOR 4.39, 95% CI 3.66-5.27 at wave 2; both *P*<.001) had the largest overall effect size.

**Conclusions:**

Suicide ideation is prevalent and significantly increasing over time in this COVID-19 pandemic era, with considerable variability between countries. Younger adults and those residing in Hong Kong carried disproportionately higher rates. Social media appears to have an increasingly detrimental association with suicide ideation, although having a stronger SOC had a larger protective effect. Policies and promotion of SOC, together with disseminating health information that explicitly tackles the infodemic’s misinformation and disinformation, may importantly reduce the rising mental health morbidity and mortality triggered by this pandemic.

## Introduction

Since the first known case was identified in Wuhan, China, the COVID-19 pandemic has led to globally significant physical and mental health sequelae [1,2] and extraordinary financial costs [3]. Inconsistent, continually evolving, and often swiftly implemented international and national response measures aimed at preventing the spread of COVID-19 have impacted all facets of society. Responses, while varied, commonly included stringent control measures such as lockdown and isolation periods, quarantine, restricted social gatherings and physical distancing, school and workplace closures, and domestic and international travel curtailments. The scale of global economic disruption from the COVID-19 pandemic has been unprecedented, resulting in countless business failures and job losses [3], despite multiple stimulus packages aimed at limiting the human and economic impacts of the pandemic [4]. Fear, anxiety, uncertainty, fatigue, together with the social and economic effects of the virus and associated countermeasures, have directly contributed to increased mental health burden [2,3]. This burden is unequally shared, disproportionately affecting vulnerable groups including young adults, students, ethnic minorities, and adults in socially or economically precarious situations [5,6]. In an effort to mitigate this mental health burden, many governments around the world have also implemented additional mental health support and financial measures [2].

The mental health burden of the COVID-19 pandemic has also been fueled by an infodemic—a rapid and far-reaching information overload, which includes misinformation and disinformation, that can serve to undermine or stymie public health responses [7-9]. The negative influence of excessive media exposure on mental health is receiving increasing attention and recognition [8-11], although its impact across the myriad of mental health and well-being domains has yet to be fully understood. In addition to national and international efforts aimed at readdressing this infodemic, such as a joint statement by the World Health Organization, the United Nations, and the United Nations Children’s Fund among others [7], it has been opined that its effect can be buffered by individuals’ and communities’ psychological resources. Family functioning, social support, social participation, trust in agencies including health care institutions, and sense of coherence (SOC) are considered to be important resistance resources [7,9,12,13]. SOC develops over the life course, and those with a stronger SOC are able to understand, handle, and make sense of a stressful situation [9,12]. This likely increases individuals’ capacities to use resistance resources to more effectively deal with the COVID-19 pandemic and associated circumstances [9,12,13]. On a population level, the infodemic is considered a major threat, with its promotion of noncompliance with public health measures; this reduces the effectiveness of these measures and ultimately enables the virus to continue to thrive [7]. On an individual level, it adds to confusion and strains mental health, already exacerbated by the pandemic. Therefore, it is essential to understand the importance of the infodemic, together with the factors and their role in mediating its effect.

Despite many national and international efforts, the global mental health burden of the COVID-19 pandemic is heavy [8], appears to be worsening [9], and is unequally shared within and between countries [8,9]. As a result, negative psychiatric and psychological responses are likely to be more prevalent, leading to poor mental health outcomes including, in the most severe cases, suicide. However, early findings from high-income and upper-middle-income countries suggest that the COVID-19 pandemic has not been associated with increases in population-level suicide rates [2]. Whether these findings remain true for lower-income countries or over longer timeframes, as the pandemic and associated global response measures continue, is open to conjecture and warrants future investigation. One mechanism for investigation is the monitoring of suicide ideation—a broad term used to describe a range of contemplations, wishes, and preoccupations with death [14]—an important risk factor for suicide [15]. Such monitoring is critical not only in alerting and informing governments and mental health agencies of a looming public health crisis but also to avert this already noted global issue with the aid of appropriate planning and prevention [16].

At the beginning of the pandemic, a cross-sectional convenience sampling study of suicide ideation among the general population across 10 countries between March 24, 2020 and April 30, 2020 (25,053 participants; 22.7% male) revealed significant differences between countries and among participants who were of younger age, male, married, and with various health beliefs [17]. That study included adults aged ≥18 years from Hong Kong (n=11,368), Brazil (n=8375), China (n=956), the United Kingdom (n=845), Turkey (n=782), the United States (n=717), the Republic of Korea (n=658), Canada (n=508), Philippines (n=454), and Macau (n=186); overall suicide ideation within the previous 2 weeks was indicated by 15.7% of participants, derived from item 9 of the Patient Health Questionnaire-9 (PHQ-9) [18]. Suicide ideation ranged from 7.6% of participants in Brazil and the United Kingdom to 24.9% of participants in the Philippines [17]. However, the reliance on convenience sampling limited the external validity of these findings, and further tracking over time would provide much needed epidemiological information.

Using an 8-country, repeated-measure, cross-sectional study design, based on representative samples of adults and including several previously surveyed countries with the same PHQ-9 measurement instrument, this investigation had 2 primary aims: (1) to estimate and compare country-specific prevalence of suicide ideation at 2 different time points, overall and by gender and age groups, and (2) to investigate the influence of sociodemographic and infodemic variables on suicide ideation. In this paper, we contextualized findings with those published elsewhere to strengthen our understanding of suicide ideation across nations and people, to grasp the effect of the infodemic, and to provide empirical evidence that will ultimately be used to save lives.

## Methods

### Study Design

This was a repeated, 8-country, cross-sectional study, with measurement wave 1 conducted from May 29, 2020 to June 12, 2020 and measurement wave 2 conducted during November 6-18, 2020.

### Participants

Study participants included adults aged ≥18 years residing in 1 of 8 countries from 4 continents (Canada, the United States, England, Switzerland, Belgium, Hong Kong, Philippines, and New Zealand) at the time of surveying.

### Primary Measure

Our primary measure, self-reported suicide ideation, was derived from item 9 of the PHQ-9 [18]. The PHQ-9 asks: “Over the past 2 weeks, how often have you been bothered by the following problems?”, with item 9 asking “Thoughts that you would be better off dead or hurting yourself in some way.” Response options include (0) not at all, (1) several days, (2) over half the days, and (3) nearly every day. Suicide ideation responses were dichotomized into indicated (combining responses 1 through 3) and otherwise (response 0) categories. The PHQ-9 is available in multiple languages and has excellent internal consistency (Cronbach α=.89) and test-retest reliability (*r*=0.84) among primary care participants [18].

### Sociodemographic, Pandemic, and Infodemic Variables

A detailed account of these variables and their definitions appears elsewhere [8]. In brief, gender identity was elicited with the following response options: male, female, another gender identity, I don't know/I prefer not to answer. Age in years was asked, with responses collapsed into the following groupings: 18-24, 25-34, 35-44, 45-54, 55-64, 65-74, and ≥75 years. The usual household composition was defined as living alone, living with others including children, or living with others but without children; being an essential worker (eg, health care and social services, law enforcement, emergency services, provider of essential goods, educational institution) was indicated by a “yes” response. Those who worked in health care and social services were further partitioned from the other essential workers. The overarching goal of this interdisciplinary and international research project was to better understand how risk information is delivered and communicated by authorities and media and how it is received, understood, and used by the public. The perceived factors and threats caused by COVID-19 that are directly related to self were investigated, together with sources and trust in information [19]. Table 1 gives the names, descriptions, and response options of all utilized pandemic- and infodemic-related variables included in the survey. At both measurement waves, the questionnaire was validated by the project collaborators, then translated and made available in the English, French, German, Italian, and Chinese languages [8].

**Table 1:** Names, descriptions, and response options for the considered variables influenced by the pandemic.

Name	Descriptions	Response options
Self-isolation/quarantine	Having experienced self-isolation/quarantine, mandatory or voluntary	Yes because of symptoms or diagnosis of COVID-19, yes for other reasons, no
Financial losses	Having experienced financial losses of any kind due to COVID-19	Yes, no, unsure/ unknown
Threat perceived for oneself and/or family	Level of threat posed by the COVID-19 perceived for oneself and/or the family	Very low, low, moderate, high, very high
Threat perceived for country and/or world	Level of threat posed by COVID-19 perceived for the country and/or the world	Very low, low, moderate, high, very high
Being a victim of stigma	Being a victim of stigma or discrimination due to COVID-19	Yes, no, decline to answer
Level of information about COVID-19	Level would you rank your level of information about COVID-19	10-point scale: 1, very low level; 1-8, otherwise; 9-10, high level; 10, very high level
Trust in authorities score	Level at which you would rank your level of trust in (1) scientists, doctors, and health experts; (2) national health organizations; (3) global health organizations; (4) government	Each response rated on a 10-point scale: 1, very low level; 10, very high level; 4 scores summed, and partitioned into approximate quartiles based on measurement wave 1-response distributions
Internet-based social media as a regular source of information	Extent that social networks (eg, Facebook, Twitter, Instagram, other networks) are used to inform yourself about COVID-19	Mainly/always, often, sometimes, not much/never
Friends/family/co-workers as a regular source of information	Extent that friends/family/co-workers are used to inform yourself about COVID-19	Mainly/always, often, sometimes, not much/never
Sense of coherence	Measured using the 3-item Sense of Coherence (SOC-3) instrument [20,21], corresponding to comprehensibility, manageability, and meaningfulness; participants were asked (1) Do you usually see a solution to problems and difficulties that other people find hopeless? (2) Do you usually feel that your daily life is a source of personal satisfaction? (3) Do you usually feel that the things that happen to you in your daily life are hard to understand?	Each with response options: no (0), yes - sometimes (1), yes - usually (2); question (3) was reverse scored; then, the 3 scores were summed and dichotomized using the threshold: weaker (summed score of 0-4) or stronger (summed score of 5-6).

### Procedure

A detailed description of the procedure and analyses involving these survey data to answer different research questions appears elsewhere [8,9]. Two polling firms, in collaboration with international partners, undertook recruitment and data collection using an online platform. Participants were randomly recruited from online panels using multiple sources, including traditional and mobile telephone methodologies (through a call center), social media (through Facebook and Instagram), and offline methods (through partner programs and campaigns such as friend recommendations). Quota sampling was used to ensure recruitment and representation of hard-to-reach groups. Once contacted and eligibility confirmed, a full explanation of the study purpose, methods of data management, and assurance of confidentiality was provided to potential participants prior to their agreement to participate in the online study. The survey was designed to take approximately 20 minutes to complete.

Selection of countries for inclusion was based on ensuring global continent diversity within a constrained budget and capturing different demographics, health systems and policies, and COVID-19 burdens and responses. Moreover, it was deemed necessary to invite country-specific lead investigators to provide context and ensure the survey was culturally fit-for-purpose. The core team came together from multiple existing professional connections, including the World Health Organization Thematic Platform for Health Emergency and Disaster Risk Management Research Network.

The implemented quota sampling was tailored for each country and based on that country’s latest available census-derived population demographics. Strata comprised of age groups (18-24 years, 25-34 years, 35-44 years, 45-54 years, 55-64 years, ≥65 years), gender (female, male), and region (which was country-specific; eg, for Canada: Ontario, Québec, British Columbia, Alberta, Manitoba/ Saskatchewan, Atlantic provinces). A 70% minimum recruitment of the estimated stratum numbers for each characteristic (age, gender, and region) was targeted in order to ensure the best possible representation in the sample. The collected data were then weighted by the population demographic distributions to reach the final representative sample.

For each country, a minimum sample target was set at 1000 adults, except for Canada (the host country of the lead investigators), which was set at 1500. Common to broad-based, multipurpose epidemiology studies, a number of core principles and pragmatic considerations were invoked in selecting these sample sizes. They include (1) a largely balanced sample size for each country so that investigations of differences between countries has maximal statistical power, (2) the power to detect differences in proportions of ≥10% or a relative risk of ≥1.2 exceeds 80% at the 2-tailed α=.05 within each country (these detectable differences are moderate to large and likely to be of clinical or meaningful significance), and (3) to maximize the number of different countries able to participate within a constrained budget.

### Statistical Analysis

Reporting of study findings was informed by the STROBE (STrengthening the Reporting of OBservational studies in Epidemiology) guidelines [22]. Participant numbers and demographics by countries and measurement waves were initially described and compared using Pearson design-based *F* tests, which also accounted for sample weightings. Next, the crude overall PHQ-9 response distributions by measurement wave were described and compared using Pearson design-based *F* tests. Age-standardized suicide ideation rates across countries and measurement waves were then determined and compared. Direct standardization was employed, with the reference population drawn from the combined wave 1 and wave 2 samples and then stratified by age groups. This derived reference population was preferred, rather than adopting any standard population, due to the particular mix of lower- and higher-income countries included within this study. Age-specific observed rates stratified by age groups were then derived for each country and measurement wave separately, and the weighted average of the stratum-specific rates, together with measures of their variability, relative to the reference population was calculated. Analysis of variance was used to compare rates between countries for each measurement wave, and 2-sample Student *t* tests were employed to test the mean difference in age-standardized rates between measurement waves by country.

Treating countries as fixed effects, a binomial regression model with an identity link function was then used to estimate and compare rates of suicide ideation indication by gender and age groups for each country and measurement wave. The identity link function was chosen as multivariable-adjusted prevalences and their differences were of primary reporting interest [23]. In this model, gender, age group, country, and measurement wave main effects were initially considered together with all their 2-factor interaction term combinations. Backward stepwise elimination of nonsignificant interaction terms followed, determined by sequentially removing nonsignificant interaction terms yielding the highest Ward type III ꭓ^2^ *P* value, to derive the final model. The main effects and interaction terms in this final model represented a baseline combination of variables.

Binomial regression models with log link function were next considered to relate sociodemographic, pandemic, and infodemic variables to suicide ideation. However, despite using different maximization techniques (such as maximum likelihood optimization and iterated, reweighted least-squares optimization of the deviance) and starting value searches, some of these models failed to converge, which is a widely recognized issue [23]. Instead, more stable logistic models were employed. For the crude analysis, each variable and its interaction with measurement wave were added and investigated in a regression model that also included the baseline combination of variables. Finally, an adjusted analysis was conducted, whereby all considered sociodemographic, pandemic, and infodemic variables together with their interaction by measurement wave were simultaneously included in a regression model that also contained the baseline combination of variables. No main effect nor interaction term variable selection was undertaken for this adjusted analysis. A direct evaluation of the final model fit was unable to be conducted as most diagnostics are unavailable when the data include survey sampling weights. Instead, an indirect evaluation was conducted whereby the final model was rerun without these weights. The Hosmer-Lemeshow goodness-of-fit test was conducted using the conventionally employed 10 partitions [24]. This was followed by an area under the receiver operating characteristic curve (AUC) analysis. AUC is frequently employed as a summary measure of a model’s predictive accuracy [25]. Adopting the recommendations of Hosmer and Lemeshow [24], an AUC of .5 suggests no discrimination, .7-.8 is considered acceptable, .8-.9 is considered excellent, and more than .9 is considered outstanding. All analyses were conducted using Stata SE version 16.0 (StataCorp, College Station, TX), accommodating the survey sampling weights and employing robust variance estimators. A 2-tailed α=.05 defined significance.

### Ethics

This study sits within a broader program of research funded by the Canadian Institutes of Health Research, reviewed and approved by the Research Ethics Board of the Centre intégré universitaire de santé et de services sociaux (CIUSSS) de l’Estrie—Center hospitalier universitaire de Sherbrooke (CHUS; Human Ethics Committee [HEC] ref: 2020-3674). Informed consent was obtained from all participants before their participation, and the collection of information was carried out confidentially. Participants were able to withdraw at any time without penalty or need for explanation. The data sets did not carry any personally identifiable information. The study complied with the ethical standards for human experimentation as established by the Helsinki Declaration and Canada’s HEC. All methods and reporting were performed in accordance with the HEC’s relevant guidelines and regulations.

## Results

### Participants

The final sample totaled 17,833 adults: 8806 (49.4%) from measurement wave 1 and 9027 (50.6%) from wave 2. Overall, 51.6% (9204/17,833) were female, and 49.2% (8769/17,833) were aged between 18 years and 44 years. The sample numbers and weighted distribution (%) of participants’ demographic characteristics by country and measurement wave appear in Table 2. Significant differences between countries were observed for age groups at measurement wave 1 (*P*<.001) and wave 2 (*P*<.001) but not for gender (*P*=.68 and *P*=.70 for measurement waves 1 and 2, respectively). Consistent with global demographic patterns, Filipino participants were younger than their non-Filipino counterparts.

**Table 2:** Participant numbers and weighted distribution (%) of their demographic characteristics by country and measurement waves 1 (surveyed between May 29, 2020 and June 12, 2020) and 2 (surveyed November 6-18, 2020).

Country		Gender^a^, n (%)		Age (years), n (%)						
		Female	Male	18-24	25-34	35-44	45-54	55-64	65-74	≥75
**Canada**										
	Wave 1 (n=1501)	723 (48.4)	772 (51.6)	163 (10.9)	247 (16.4)	243 (16.2)	269 (17.9)	262 (17.5)	246 (16.4)	72 (4.8)
	Wave 2 (n=2004)	963 (48.3)	1031 (51.7)	218 (10.9)	329 (16.4)	324 (16.2)	359 (17.9)	350 (17.5)	340 (17.0)	84 (4.2)
**United States**										
	Wave 1 (n=1065)	516 (48.5)	548 (51.5)	59 (5.5)	226 (21.2)	191 (17.9)	204 (19.1)	189 (17.8)	146 (13.7)	50 (4.7)
	Wave 2 (n=1003)	478 (48.1)	517 (51.9)	81 (8.0)	187 (18.7)	180 (17.9)	192 (19.1)	178 (17.8)	71 (7.1)	114 (11.4)
**England**										
	Wave 1 (n=1041)	508 (48.8)	532 (51.2)	116 (11.1)	181 (17.4)	170 (16.3)	186 (17.9)	151 (14.5)	190 (18.3)	47 (4.5)
	Wave 2 (n=1000)	487 (48.8)	511 (51.2)	111 (11.1)	174 (17.4)	163 (16.3)	179 (17.9)	145 (14.5)	192 (19.2)	35 (3.5)
**Belgium**										
	Wave 1 (n=1015)	494 (48.6)	521 (51.4)	63 (6.2)	208 (20.5)	139 (13.7)	210 (20.7)	171 (16.9)	186 (18.3)	37 (3.7)
	Wave 2 (n=1014)	489 (48.4)	520 (51.6)	57 (5.6)	215 (21.2)	118 (11.6)	228 (22.5)	161 (15.9)	197 (19.4)	38 (3.7)
**Switzerland**										
	Wave 1 (n=1002)	478 (47.7)	523 (52.3)	95 (9.5)	144 (14.4)	138 (13.8)	177 (17.6)	239 (23.9)	160 (16.0)	48 (4.8)
	Wave 2 (n=1000)	477 (47.8)	522 (52.2)	95 (9.5)	144 (14.4)	138 (13.8)	176 (17.6)	171 (17.1)	226 (22.6)	49 (4.9)
**Hong Kong**										
	Wave 1 (n=1140)	513 (45.1)	626 (54.9)	108 (9.5)	196 (17.2)	206 (18.1)	218 (19.1)	202 (17.7)	200 (17.5)	10 (0.9)
	Wave 2 (n=1002)	451 (45.0)	550 (55.0)	95 (9.5)	172 (17.2)	181 (18.1)	192 (19.1)	177 (17.7)	171 (17.1)	13 (1.3)
**Philippines**										
	Wave 1 (n=1041)	510 (49.4)	522 (50.6)	224 (21.6)	260 (25.0)	209 (20.1)	162 (15.5)	106 (10.2)	63 (6.1)	17 (1.6)
	Wave 2 (n=1003)	489 (49.3)	503 (50.7)	216 (21.6)	251 (25.0)	201 (20.1)	156 (15.5)	126 (12.5)	47 (4.7)	6 (0.6)
**New Zealand**										
	Wave 1 (n=1001)	484 (48.6)	512 (51.4)	122 (12.2)	184 (18.4)	163 (16.3)	175 (17.5)	157 (15.7)	149 (14.9)	50 (5.0)
	Wave 2 (n=1001)	484 (48.6)	513 (51.4)	122 (12.2)	184 (18.4)	163 (16.3)	175 (17.5)	157 (15.7)	138 (13.8)	61 (6.1)

^a^25 participants at the measurement wave 1 and 42 participants at measurement wave 2 did not identify as being female (F) or male (M) or preferred not to answer this question, so had their gender set to missing.

### Suicide Ideation

#### Overall Rates

At measurement wave 1, 75.8% (6674.9/8806) of participants had no thoughts of being better off dead or hurting themselves in some way over the previous 2 weeks, whereas 14.7% (1247.9/8806) had these thoughts on several days, 6.4% (565.9/8806) had these thoughts over half the days, and 3.6% (317.3/8806) reported having these thoughts nearly every day. Approximately 5 months later, at the second measurement wave, 72.5% (6541.3/9027) of participants had no thoughts of being better off dead or hurting themselves in some way over the previous 2 weeks, 15.3% (1379.3/9027) had these thoughts on several days, 7.8% (706.6/9027) had these thoughts over half the days, and 4.4% (399.8/9027) reported having these thoughts nearly every day. These response distributions were significantly different between measurement waves (*P*<.001), with a 3.3% decrease in those having no thoughts of death of harm over the previous 2 weeks at measurement wave 2 compared with wave 1 and concomitantly, a 2.2% increase in those reporting having these thoughts nearly every day or over half the days.

#### Age-Standardized Rates

**Figure 1:**
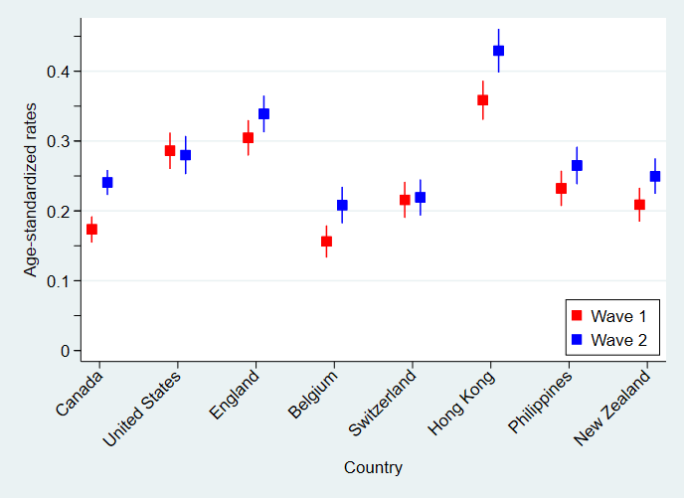
Age-standardized rates and associated 95% CIs of suicide ideation by country for measurement waves 1 (surveyed between May 29, 2020 and June 12, 2020) and 2 (surveyed November 6-18, 2020).

Figure 1 presents the age-standardized rates of the dichotomized suicide ideation variable across countries for measurement waves 1 and 2. Table S1 in Multimedia Appendix 1 gives the combined total age distribution of participants over measurement waves and countries used as the reference population for these age standardization calculations. Perusal of Figure 1 suggests that important differences in self-reported suicide ideation exist between countries and measurement waves. Analysis of variance confirmed this, with significant differences in age-standardized rates found between countries at measurement wave 1 (*P*<.001) and wave 2 (*P*<.001). Rates in Hong Kong participants, for instance, were significantly higher than all other countries at both measurement waves (all *P*<.001, except for *P*=.006 when compared with England participants at measure wave 1). When comparing age-standardized rates of suicide ideation between measurement waves 1 and wave 2, significant increases were observed for participants in Canada (mean difference .067, 95% CI .041-.094; *P*<.001), Belgium (mean difference .052, 95% CI .017-.087; *P*=.004), Hong Kong (mean difference .071, 95% CI .029-.113; *P*<.001), and New Zealand (mean difference .041, 95% CI .006-.076; *P*=.02) but not for participants in the United States (*P*=.75), England (*P*=.07), Switzerland (*P*=.86), or the Philippines (*P*=.08).

#### Age- and Gender-Adjusted Comparison of Suicide Ideation Between Countries

**Figure 2:**
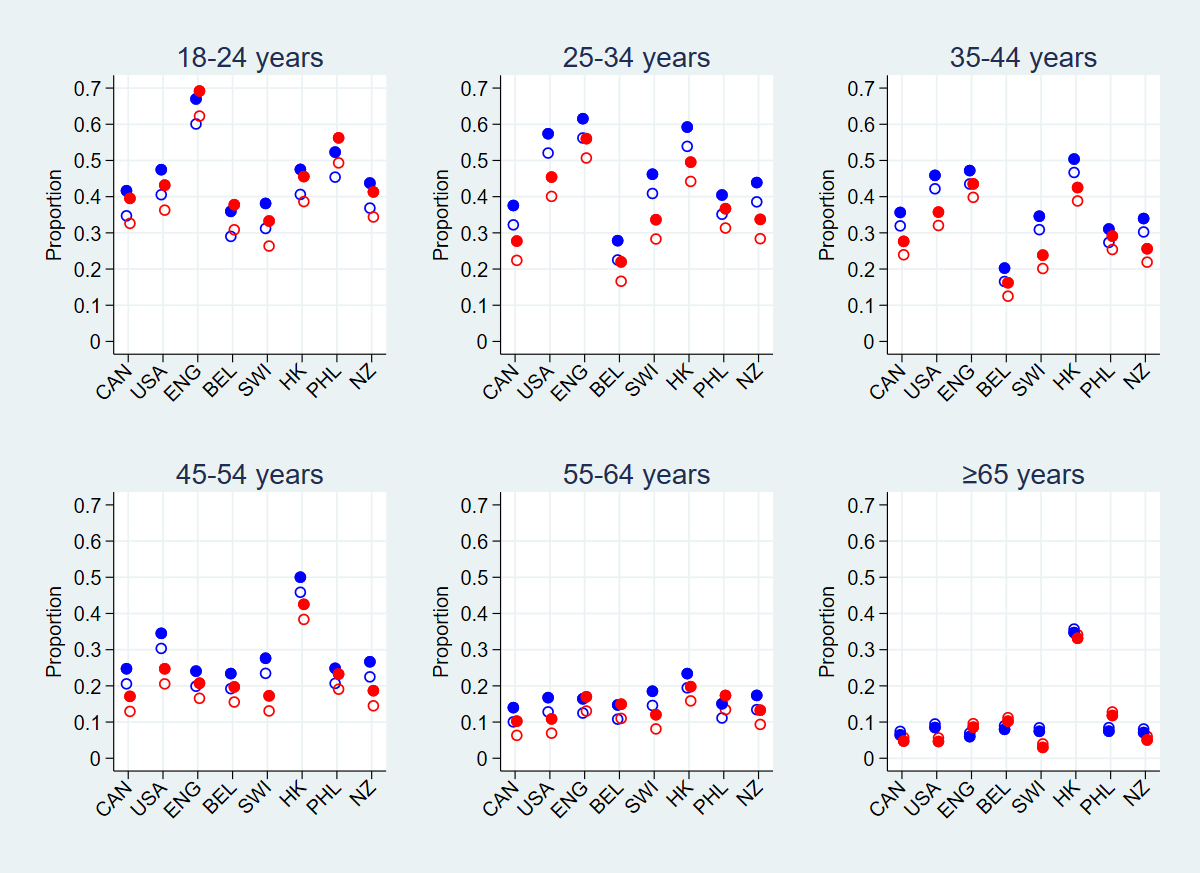
Estimated proportion of participants self-reporting suicide ideation by country and measurement waves 1 and 2 stratified by age group, derived from the binomial regression model including age group, gender, country, measurement wave, and the measurement wave × age group, country × age group, country × gender, and age group × gender interactions. Females are denoted by red, males by blue, measurement wave 1 with hollow circles, and measurement wave 2 with solid circles. BEL: Belgium; CAN: Canada; ENG: England; HK: Hong Kong; NZ: New Zealand; PHL: Philippines; SWI: Switzerland; USA: United States.

As a result of the relatively small number of participants within the ≥75-year age group (see Table 1 and Multimedia Appendix 1, Table S1), they were combined with those aged 65-74 years to form the ≥65-year age category used henceforth. Initially, the statistical model of the binary measure of suicide ideation included the main effects of gender, age group, country, and measurement wave, together with all 2-factor interactions. However, the measurement wave × gender (step 1, *P*=.46) and measurement wave × country (step 2, *P*=.12) interaction terms were nonsignificant and were thus removed, leaving a model that included gender (*P*=.44), age group (*P*<.001), country (*P*<.001), measurement wave (*P*=.003), age group × gender (*P*<.001), country × gender (*P*=.003), age group × country (*P*<.001), and age group × measurement wave (*P*=.001). Due to its significant interactions with age group and country, the gender main effect was retained within the final model, despite not having a significant *P* value. Figure 2 and Table S2 in Multimedia Appendix 1 provide the estimated proportions of suicide ideation indication, together with associated 95% CIs, derived from this final model. These estimated proportions ranged from .040 (95% CI .010-.069) in measurement wave 1 and .030 (95% CI .001-.059) in wave 2 among women aged ≥65 years from Switzerland to .623 (95% CI .555-.690) in wave 1 and .692 (95% CI .618-.765) in wave 2 among women aged 18-24 years from England; see Table S2 in Multimedia Appendix 1.

Overall, among women, those aged 18-24 years had the highest estimated proportion of indications at both measurement waves (mean .389 and .458, respectively) and the greatest increase between measurement waves (mean change .069). Both the proportion of indications and the difference between measurement waves dampened with increasing age group. For men, those aged 25-34 years had the highest estimated indication proportion at both measurement waves (mean .414 and .468, respectively), somewhat higher than for those aged 18-24 years (mean .398 and .467, respectively). However, similar to female participants, the greatest increase in suicide ideation indications between measurement waves (mean change .069) occurred for those aged 18-24 years, and the proportion of indications and difference between measurement waves dampened with increasing age group; see Figure 2 and Table S2 in Multimedia Appendix 1. Notable in Figure 2 and Table S2 in Multimedia Appendix 1 are the relatively high proportions of participants experiencing suicide ideation from Hong Kong across all age groups, especially among those aged 45-54 years or ≥65 years.

#### Factors Affecting Suicide Ideation

The weighted frequency distributions of the potential risk and protective COVID-19–related factors for suicide ideation indication by measurement wave are presented in Table S3 in Multimedia Appendix 1. Logistic regression–estimated crude (OR) and adjusted (AOR) odds ratios and accompanying 95% CIs of suicide ideation associated with these factors by measurement wave appear in Table 3. The crude ORs were adjusted by gender, age group, country, and measurement wave main effects together with the age group × gender, country × gender, age group × country, and age group × measurement wave interaction terms identified in the previous section. In these analyses, both the main effect and interaction by measurement wave terms were significant for variables corresponding to self-isolation/quarantine (*P*<.001 and *P*=.02, respectively), financial losses (*P*<.001 and *P*=.003, respectively), and threat perceived for oneself and/or family (*P*<.001 and *P*=.008, respectively). However, significant main effect and nonsignificant interactions by measurement wave terms were identified for variables corresponding to being an essential worker (*P*<.001 and *P*=.66, respectively), being a victim of stigma (*P*<.001 and *P*=.09, respectively), trust in authorities score (*P*<.001 and *P*=.49, respectively), internet-based social media as a regular source of information (*P*<.001 and *P*=.13, respectively), friends/family/co-worker as a regular source of information (*P*<.001 and *P*=.33, respectively), and SOC (*P*<.001 and *P*=.24, respectively). This implies that these variables have a significant relationship with suicide ideation, which did not change between measurement waves. For the remaining variables, no significant relationships were observed.

**Table 3:** Estimated crude and adjusted odds ratios (ORs) and associated 95% CIs of suicide ideation associated with potential risk and protective COVID-19 related factors by measurement wave for the logistic model that included the entire sample over both measurement waves.

Factors		Crude OR (95% CI)^a^		Adjusted OR (95% CI)^b^	
		Wave 1	Wave 2	Wave 1	Wave 2
**Household composition**					
	Alone	1 (reference)	1 (reference)	1 (reference)	1 (reference)
	With children	0.93 (0.78-1.11)	1.02 (0.85-1.23)	1.03 (0.83-1.27)	1.05 (0.85-1.29)
	With others	0.89 (0.75-1.06)	0.88 (0.74-1.05)	0.98 (0.81-1.20)	0.94 (0.77-1.13)
**Essential worker**					
	No	1 (reference)	1 (reference)	1 (reference)	1 (reference)
	Yes: health	1.72 (1.45-2.04)	1.60 (1.33-1.91)	1.60 (1.31-1.96)	1.36 (1.10-1.69)
	Yes: other	1.43 (1.24-1.65)	1.32 (1.14-1.52)	1.29 (1.09-1.53)	1.22 (1.03-1.44)
**Self-isolation/quarantine**					
	No	1 (reference)	1 (reference)	1 (reference)	1 (reference)
	Yes: case/symptom-free	1.16 (1.01-1.32)	1.42 (1.24-1.63)	1.08 (0.93-1.26)	1.23 (1.05-1.44)
	Yes: case or symptoms	3.16 (2.64-3.77)	2.84 (2.42-3.34)	2.39 (1.95-2.93)	1.91 (1.58-2.32)
**Financial losses**					
	No	1 (reference)	1 (reference)	1 (reference)	1 (reference)
	Yes	1.39 (1.23-1.57)	1.82 (1.62-2.06)	1.09 (0.95-1.25)	1.40 (1.22-1.60)
	Unsure/unknown	1.95 (1.56-2.43)	2.85 (2.15-3.78)	1.79 (1.30-2.47)	2.42 (1.50-3.91)
**Threat perceived for oneself and/or family**					
	High	1.84 (1.64-2.07)	1.47 (1.31-1.66)	1.66 (1.44-1.90)	1.31 (1.14-1.51)
	Otherwise	1 (reference)	1 (reference)	1 (reference)	1 (reference)
**Threat perceived for country and/or world**					
	High	1.00 (0.88-1.13)	0.91 (0.80-1.04)	0.85 (0.73-0.98)	0.82 (0.70-0.96)
	Otherwise	1 (reference)	1 (reference)	1 (reference)	1 (reference)
**Being a victim of stigma**					
	No	1 (reference)	1 (reference)	1 (reference)	1 (reference)
	Yes	3.34 (2.88-3.87)	3.83 (3.27-4.49)	2.58 (2.17-3.06)	2.74 (2.26-3.31)
	Decline to answer	1.64 (1.37-1.95)	2.24 (1.73-2.89)	1.15 (0.90-1.45)	1.23 (0.78-1.95)
**Level of information about COVID-19**					
	High	1 (reference)	1 (reference)	1 (reference)	1 (reference)
	Otherwise	0.96 (0.85-1.08)	0.98 (0.86-1.11)	0.91 (0.79-1.06)	0.97 (0.83-1.13)
**Trust in authorities score**					
	Q1 (low)	1.39 (1.19-1.63)	1.39 (1.19-1.63)	1.56 (1.29-1.89)	1.40 (1.15-1.71)
	Q2	1.15 (0.98-1.36)	1.27 (1.08-1.50)	1.29 (1.06-1.57)	1.34 (1.10-1.63)
	Q3	0.96 (0.81-1.13)	1.11 (0.95-1.31)	1.06 (0.87-1.28)	1.09 (0.90-1.32)
	Q4 (high)	1 (reference)	1 (reference)	1 (reference)	1 (reference)
**Internet-based social media** **as a regular source of information**					
	Often/always	1.35 (1.19-1.52)	1.54 (1.35-1.75)	1.11 (0.96-1.30)	1.47 (1.25-1.72)
	Sometimes/never	1 (reference)	1 (reference)	1 (reference)	1 (reference)
**Friends/family/co-workers as a regular source of information**					
	Often/always	1.23 (1.10-1.38)	1.14 (1.01-1.28)	1.06 (0.92-1.23)	0.96 (0.83-1.11)
	Sometimes/never	1 (reference)	1 (reference)	1 (reference)	1 (reference)
**Sense of coherence**					
	Stronger (5-6)	1 (reference)	1 (reference)	1 (reference)	1 (reference)
	Weaker (0-4)	3.96 (3.36-4.67)	4.56 (3.85-5.40)	3.80 (3.18-4.55)	4.39 (3.66-5.27)

^a^Adjusted for gender, age group, country, measurement wave, age group × gender, country × gender, age group × country, and age group × measurement wave.

^b^Adjusted for all variables included within this table, together with their interaction by measurement wave and the main effect and interactions terms included within the crude analysis.

When considered together in the adjusted analysis, the patterns of associations remained the same except that “friends/family/co-worker as a regular source of information” was no longer significant (main effect *P*=.38; interaction *P*=.32), “threat perceived for country and/or world” had a significant main effect (*P*=.03) but nonsignificant interaction (*P*=.80), and “internet-based social media as a regular source of information” had a nonsignificant main effect (*P*=.16) but significant interaction (*P*=.01), so that frequent users of social media were associated with significantly higher suicide ideation than infrequent users at measurement wave 2 (*P*<.001). Based on the magnitude of the *z* score, having a weaker SOC was associated with the highest likelihood of suicide ideation among the factors considered (AOR 3.80, 95% CI 3.18-4.55; *P*<.001 at wave 1, and AOR 4.39, 95% CI 3.66-5.27; *P*<.001 at wave 2), followed by identifying as being a victim of stigma (AOR 2.58, 95% CI 2.17-3.06; *P*<.001 at wave 1 and AOR 2.74, 95% CI 2.26-3.31; *P*<.001 at wave 2). Hong Kong participants were the most likely to report a weaker SOC. At measurement wave 1, 90.0% (1025.9/1140) of Hong Kong participants reported a weaker SOC compared with 66.9% (5130.4/7666) of participants in the other countries, while for measurement wave 2, the percentages were 90.3% (905.0/1002) and 67.3% (5402.7/8025), respectively.

In the adjusted model, re-analyzed without survey sampling weights, the Hosmer-Lemeshow goodness-of-fit was not significant (*P*=.38), and the AUC was .803 (95% CI .795-.810), which is on the cusp between acceptable and excellent. This indirect evidence suggests that the final model has adequate fit.

## Discussion

### Principal Findings

Suicide ideation is prevalent and increased significantly over time in the COVID-19 pandemic. Overall, 24.2% and 27.5% of adults aged ≥18 years reported at least one such thought at measurement waves 1 and 2, respectively. This is substantially higher than the 15.5% reported from a study undertaken approximately 2 months earlier than measurement wave 1 [17]. However, like the previous study, there was considerable variability between countries in the age-standardized rates of suicide ideation at measurement wave 1, ranging from 15.6% (95% CI 13.3%-17.9%) in Belgium to 35.8% (95% CI 33.0%-39.7%) in Hong Kong, and measurement wave 2, ranging from 20.8% (95% CI 18.2%-23.5%) in Belgium to 42.9% (95% CI 39.8%-46.1%) in Hong Kong. This significant increase in overall rates between studies likely reflects people’s significant psychological deterioration over time, as observed within this study; the different composition of countries investigated; and the fundamentally different sampling strategies. Reliance on convenience sampling, particularly in surveys of mental health (where individuals with existing or severe mental illness are less likely to participate), is prone to substantial bias [26].

Hong Kong adults had demonstratively higher rates of suicide ideation than their counterparts in the comparator countries. This may reflect the cumulative effects of COVID-19 together with the political instability and social unrest of that time, which included the 2019-2020 Hong Kong protests [27]. These protests and the accompanying social unrest may have also directly contributed to the Hong Kong participants having consistently lower SOC levels (the greatest protective factor, over and above age and gender, against suicide ideation observed in this study) compared with the other investigated countries. Although the lower SOC levels may also be cultural, or even simply arise from different understandings of the questions in different cultures, it behooves further investigation.

Previously, a population-based prospective cohort of adults aged ≥18 years identified a major mental health burden during a time of social unrest in Hong Kong, although suicide ideation was identified in only 4.3% of their respondents using the same PHQ-9 instrument [28]. Also using the PHQ-9 item 9, another population-representative sample of Hong Kong residents aged ≥15 years conducted in July 2019 reported that 9.1% of their participants had suicide ideation [29]. These rates are significantly less than the 22.0% reported in a multinational study of suicide ideation [17] and the age-standardized rate of 35.8% reported here. Although the Hong Kong rate of suicide increased with the severe acute respiratory syndrome (SARS) outbreak in 2003, particularly among older adults, it has stabilized since to around 13.0/100,000 people per year [30]. This 2019 age-standardized rate for Hong Kong is higher than that in Canada or New Zealand, both estimated at 10.3/100,000 people per year, but less than that in the United States or Belgium, estimated at 14.5/100,000 and 13.9/100,000 people per year, respectively [30,31]. Thus, it could be argued that suicide and suicide ideation in Hong Kong may reflect culturally traditional patterns rather than a result of lower SOC [32].

Both age and gender were associated with suicide ideation. Apart from Hong Kong participants, rates decreased as age groups increased, a finding consistent with the literature [14,33]. Among those aged 18-24 years, 55-64 years, or ≥65 years, women in England, Belgium, and the Philippines had higher estimated suicide ideation rates than men. Interestingly, according to the Worldometer [34], cumulative COVID-19 death rates for England (60.6 and 115.6 per 100,000 in June 2020 and November 2021, respectively) and Belgium (82.0 and 125.1 per 100,000 in June 2020 and November 2021, respectively) were highest among the countries investigated here, although the Philippines ranked sixth among the 8 countries. Women may have been differentially impacted or burdened by the relatively high mortality rates in England and Belgium, and the cultural expectations of Filipino women could contribute to these observations.

In all other countries and age groups, the reverse pattern was observed, with men having higher estimated rates than women. Arguably, apart from those aged 25-34 years, these gender differences were relatively small compared to the variations across age groups, countries, and measurement waves—suggesting that this extraordinary pandemic effect transcends previously documented gender differences. It is noteworthy that the reported results from the general population 2015 National Survey on Drug Use and Health in the United States showed similar variability between gender across age groups [33]. Strikingly and perhaps unsurprisingly, the factor with the most protective effect against suicide ideation, over age and gender, was having a stronger SOC. An increased SOC has previously been associated with lower rates of suicide ideation and attempts of suicide [35-37]. It also has been shown to be associated with lower rates of common psychopathological symptoms in this pandemic [8,9] and thus arguably, is an important underestimated resource in minimizing the COVID-19 psychosocial impact [8]. Health promotion strategies that strengthen SOC may provide a useful protective mechanism to assuage people’s mental health burdens [38].

Although having a smaller effect size, another key finding was the rise and significance of internet-based social media as a regular source of information associated with suicide ideation in the adjusted analyses. In the first measurement wave, those who often or always used social networks as a regular source of information had an AOR of 1.11 (95% CI 0.96-1.30) compared with those who sometimes or never used social networks, a nonsignificant difference (*P*=.16). However, by measurement wave 2, the AOR had increased to 1.47 (95% CI 1.25-1.72), a difference that was significant (*P*<.001). Although social media can have a crucial role in disseminating health information and tackling infodemics and misinformation [39], frequent exposure to social media has been associated with mental health problems during the COVID-19 outbreak [40]. Interestingly, the deterioration in mental health associated with frequent social media use over the course of this pandemic has also been previously observed in 2 studies from China [40,41].

### Strengths and Limitations

Although having salient strengths, such as the relatively large sample size, the timeliness of the recruitment and analysis, the spread of participants across 8 countries and 4 continents, and the repeated nature of the survey using consistent and psychometrically robust instruments, this study also has limitations. Arguably, the greatest potential weakness is the sampling mechanism and associated unmeasurable nonsampling bias. The employed sampling frame is more opaque than traditional or conventionally used frames. Participants were nonetheless randomly recruited from panels developed using multiple online and offline sources. Moreover, quota targeting sampling together with survey sampling weights were used to ensure approximately representative samples. In designing, attracting funding for, securing ethics for, and implementing this study, there was a pragmatic requirement to maximize expedited data collection, sample frame availability, cost effectiveness, and international reach while minimizing nonsampling bias. The selected approach sought to optimize these requirements and yield reliable and robust research data. However, despite considerable efforts undertaken to derive representative samples, some population groups may be underrepresented, including people without internet access or those with lower literacy levels [26]. In addition, people living with existing disabilities (including mental illness) are less likely to participate in online surveys compared with those without such disabilities and illnesses [26,42]. Although sampling weightings were adopted to ameliorate this effect, these adjustments may miss crucial elements of bias and cannot account for groups not included within the surveys.

Another potential weakness is the repeated cross-sectional rather than longitudinal study design, which negates any causal assertions. Moreover, individual participant changes over time cannot be investigated. However, assuming the sampling strategy and uptake remain consistent, valid population-level trend analyses can be conducted [43]. A careful statistical approach was employed here, which sought to disentangle systematic patterns from sampling variability, to investigate population-level, time-varying changes between measurement waves. Furthermore, ORs were reported rather than prevalence ratios (PRs). As suicide ideation was relatively common in this study, these ORs may overestimate their respective PRs and should not be interpreted as measures of relative risk [44]. Binomial regression models estimating PRs were initially entertained, but convergence issues arose. Instead, the more stable logistic models were employed without issue [23]. However, regardless of the regression model ultimately employed, the reported results may suffer from residual or unmeasured confounding effects [45]. For example, questions on the availability of face masks and protective clothing and the market flooding of ineffective counterfeit versions in Hong Kong [46] were not asked but are likely to contribute to poor mental health and suicide ideation of its people. Unmeasured confounding variables can result in substantial bias in the estimated exposure-outcome AOR, particularly if it is uncorrelated with the measured explanatory variables. Study replication using different suites of variables is needed to understand its effect. Another potential limitation is the PHQ-9 item 9 measure itself. It has been widely used and endorsed as a single measure to assess the prevalence of suicide ideation in research studies [47]. However, this stance is not uniformly shared, with some regarding it as an insufficient assessment tool for suicide risk and suicide ideation [48]. The lack of a universally accepted consistent definition of suicide ideation leads to ongoing challenges for researchers and others [14] and makes direct study comparisons difficult.

### Conclusion

Globally, the COVID-19 pandemic era represents an extraordinary time for all societies, presenting extraordinary challenges and posing extraordinary and worsening mental health burdens on people. The high and increased rates of suicide ideation reported by participants across 8 countries in 4 continents reflect the cumulative effects of this pandemic and its associated burdens. Young adults and those in Hong Kong, in particular, have been deeply affected. SOC appears to be a potent protective force. A health promotion approach using a salutogenic orientation aimed at strengthening SOC may offer a new perspective for reducing suicide ideation. Moreover, with social media appearing to have an increasingly negative influence, it is critical for countries and health agencies to squarely redress rampant misinformation and disinformation communications. Suicide ideation is an important mental health indictor and risk factor for completed suicides. Policies and promotion of SOC, together with dissemination of health information that explicitly tackles the infodemic’s misinformation and disinformation, may importantly contribute to reducing the rising mental health morbidity and mortality triggered by this pandemic.

## Appendix

Multimedia Appendix 1 Tables S1, S2, and S3.

## Abbreviations

AOR: adjusted odds ratio
AUC: area under the receiver operating characteristic curve
CHUS: Center hospitalier universitaire de Sherbrooke
CIUSSS: Centre intégré universitaire de santé et de services sociaux
HEC: Human Ethics Committee
OR: odds ratio
PHQ-9: Patient Health Questionnaire-9
PR: prevalence ratio
SARS: severe acute respiratory syndrome
SOC: sense of coherence
STROBE: STrengthening the Reporting of OBservational studies in Epidemiology
