# Supplementary material for: Patterns of Suicide Ideation Across Eight Countries in Four Continents During the COVID-19 Pandemic Era: Repeated Cross-sectional Study
Source: JMIR Public Health Surveill. 2022 Jan 17;8(1):e32140. doi: 10.2196/32140 (PMC8765348; doi:10.2196/32140)
Supplement: Multimedia Appendix 1 [file publichealth_v8i1e32140_app1.pdf]

Supplementary materials for:

Patterns of suicide ideation across eight countries in four continents during the COVID-19 pandemic era: a repeated cross-sectional study

---

Table S1. Age distribution of participants combined over measurement waves and countries used for age standardisation calculations as the reference population.

Age group (years)	Sample numbers	Proportion
18-24	1,946.01	0.10912
25-34	3,302.82	0.18521
35-44	2,927.15	0.16414
45-54	3,257.85	0.18269
55-64	2,945.94	0.16520
65-74	2,721.41	0.15261
≥75	731.82	0.04104
Total	17,833.00	1.00000

Table S2. Estimated proportion and associated 95% confidence intervals (CIs) of participants self-reporting suicide ideation across countries for measurement waves 1 and 2 derived from the binomial regression model including age, gender, countries, measurement wave, and the measurement wave×age, country×age, country×gender, and age×gender interactions presented by country, and partitioned by gender and age groups.

	**Measurement wave 1**		**Measurement wave 2**	
	Proportion	(95% CI)	Proportion	(95% CI)
**Canada**				
Female				
18-24 years	0.326	(0.260, 0.392)	0.395	(0.332, 0.458)
25-34 years	0.224	(0.173, 0.275)	0.277	(0.225, 0.330)
35-44 years	0.239	(0.184, 0.295)	0.276	(0.218, 0.334)
45-45 years	0.129	(0.085, 0.174)	0.171	(0.122, 0.220)
55-64 years	0.063	(0.024, 0.102)	0.103	(0.059, 0.146)
≥65 years	0.057	(0.030, 0.085)	0.047	(0.019, 0.076)
Male				
18-24 years	0.347	(0.279, 0.415)	0.416	(0.355, 0.477)
25-34 years	0.322	(0.267, 0.378)	0.376	(0.319, 0.432)
35-44 years	0.319	(0.263, 0.376)	0.356	(0.299, 0.414)
45-45 years	0.206	(0.158, 0.253)	0.247	(0.197, 0.298)
55-64 years	0.101	(0.062, 0.139)	0.140	(0.099, 0.180)
≥65 years	0.074	(0.043, 0.105)	0.064	(0.033, 0.096)
**USA**				
Female				
18-24 years	0.363	(0.273, 0.453)	0.432	(0.341, 0.523)
25-34 years	0.401	(0.343, 0.458)	0.454	(0.395, 0.513)
35-44 years	0.320	(0.262, 0.379)	0.357	(0.297, 0.418)
45-45 years	0.206	(0.153, 0.258)	0.247	(0.194, 0.301)
55-64 years	0.069	(0.037, 0.102)	0.109	(0.070, 0.147)
≥65 years	0.056	(0.029, 0.083)	0.046	(0.020, 0.073)
Male				
18-24 years	0.405	(0.310, 0.500)	0.475	(0.381, 0.568)
25-34 years	0.521	(0.463, 0.579)	0.574	(0.515, 0.633)
35-44 years	0.422	(0.360, 0.483)	0.459	(0.396, 0.521)
45-45 years	0.303	(0.249, 0.358)	0.345	(0.291, 0.399)
55-64 years	0.128	(0.086, 0.171)	0.168	(0.123, 0.212)
≥65 years	0.095	(0.059, 0.131)	0.085	(0.049, 0.120)
**England**				
Female				
18-24 years	0.623	(0.555, 0.690)	0.692	(0.618, 0.765)
25-34 years	0.507	(0.447, 0.567)	0.560	(0.500, 0.620)
35-44 years	0.398	(0.339, 0.458)	0.435	(0.375, 0.496)
45-45 years	0.166	(0.117, 0.215)	0.207	(0.157, 0.258)
55-64 years	0.131	(0.085, 0.176)	0.170	(0.122, 0.218)
≥65 years	0.096	(0.059, 0.132)	0.086	(0.052, 0.119)
Male				
18-24 years	0.601	(0.530, 0.672)	0.670	(0.595, 0.745)
25-34 years	0.562	(0.499, 0.625)	0.615	(0.553, 0.678)
35-44 years	0.436	(0.375, 0.495)	0.472	(0.412, 0.532)
45-45 years	0.199	(0.148, 0.250)	0.241	(0.190, 0.291)

55-64 years	0.125	(0.078, 0.172)	0.164	(0.006, 0.212)
≥65 years	0.070	(0.037, 0.102)	0.060	(0.029, 0.091)
**Belgium**				
Female				
18-24 years	0.309	(0.214, 0.403)	0.378	(0.282, 0.474)
25-34 years	0.166	(0.118, 0.215)	0.220	(0.171, 0.269)
35-44 years	0.125	(0.076, 0.174)	0.162	(0.112, 0.213)
45-45 years	0.155	(0.107, 0.204)	0.197	(0.147, 0.247)
55-64 years	0.110	(0.070, 0.150)	0.149	(0.107, 0.191)
≥65 years	0.112	(0.076, 0.148)	0.102	(0.067, 0.138)
Male				
18-24 years	0.290	(0.187, 0.394)	0.359	(0.257, 0.462)
25-34 years	0.225	(0.172, 0.278)	0.278	(0.225, 0.332)
35-44 years	0.166	(0.107, 0.224)	0.203	(0.144, 0.261)
45-45 years	0.192	(0.146, 0.239)	0.234	(0.187, 0.281)
55-64 years	0.108	(0.063, 0.153)	0.147	(0.102, 0.192)
≥65 years	0.090	(0.058, 0.121)	0.080	(0.049, 0.111)
**Switzerland**				
Female				
18-24 years	0.264	(0.199, 0.328)	0.333	(0.262, 0.404)
25-34 years	0.283	(0.224, 0.342)	0.336	(0.277, 0.396)
35-44 years	0.201	(0.150, 0.253)	0.239	(0.183, 0.294)
45-45 years	0.131	(0.082, 0.179)	0.172	(0.121, 0.224)
55-64 years	0.081	(0.040, 0.122)	0.120	(0.077, 0.163)
≥65 years	0.040	(0.010, 0.069)	0.030	(0.001, 0.059)
Male				
18-24 years	0.312	(0.242, 0.382)	0.381	(0.307, 0.455)
25-34 years	0.409	(0.347, 0.471)	0.462	(0.399, 0.525)
35-44 years	0.309	(0.252, 0.366)	0.346	(0.287, 0.405)
45-45 years	0.235	(0.183, 0.286)	0.276	(0.223, 0.329)
55-64 years	0.146	(0.097, 0.195)	0.185	(0.135, 0.235)
≥65 years	0.084	(0.052, 0.117)	0.074	(0.043, 0.106)
**Hong Kong**				
Female				
18-24 years	0.386	(0.316, 0.456)	0.455	(0.381, 0.530)
25-34 years	0.442	(0.389, 0.496)	0.495	(0.440, 0.550)
35-44 years	0.388	(0.332, 0.444)	0.425	(0.367, 0.483)
45-45 years	0.384	(0.324, 0.443)	0.425	(0.365, 0.486)
55-64 years	0.159	(0.103, 0.214)	0.198	(0.142, 0.253)
≥65 years	0.341	(0.274, 0.409)	0.331	(0.264, 0.399)
Male				
18-24 years	0.406	(0.330, 0.482)	0.475	(0.398, 0.553)
25-34 years	0.539	(0.481, 0.597)	0.592	(0.533, 0.652)
35-44 years	0.467	(0.408, 0.525)	0.504	(0.444, 0.563)
45-45 years	0.459	(0.397, 0.521)	0.500	(0.438, 0.563)
55-64 years	0.195	(0.140, 0.249)	0.234	(0.181, 0.287)
≥65 years	0.357	(0.293, 0.421)	0.347	(0.283, 0.412)
**Philippines**				
Female				
18-24 years	0.493	(0.427, 0.560)	0.562	(0.492, 0.633)
25-34 years	0.313	(0.255, 0.372)	0.367	(0.308, 0.425)

35-44 years	0.254	(0.198, 0.310)	0.291	(0.233, 0.349)
45-45 years	0.191	(0.126, 0.255)	0.233	(0.166, 0.299)
55-64 years	0.134	(0.066, 0.202)	0.174	(0.103, 0.244)
≥65 years	0.128	(0.051, 0.205)	0.118	(0.042, 0.194)
Male				
18-24 years	0.454	(0.392, 0.516)	0.523	(0.459, 0.587)
25-34 years	0.351	(0.288, 0.414)	0.405	(0.342, 0.468)
35-44 years	0.273	(0.211, 0.336)	0.311	(0.247, 0.374)
45-45 years	0.207	(0.139, 0.274)	0.248	(0.180, 0.317)
55-64 years	0.111	(0.050, 0.173)	0.151	(0.087, 0.214)
≥65 years	0.085	(0.016, 0.153)	0.075	(0.008, 0.142)
**New Zealand**				
Female				
18-24 years	0.344	(0.275, 0.413)	0.413	(0.339, 0.487)
25-34 years	0.284	(0.226, 0.341)	0.337	(0.280, 0.395)
35-44 years	0.219	(0.164, 0.274)	0.256	(0.199, 0.314)
45-45 years	0.145	(0.097, 0.193)	0.187	(0.134, 0.240)
55-64 years	0.093	(0.049, 0.138)	0.133	(0.086, 0.179)
≥65 years	0.060	(0.031, 0.090)	0.051	(0.022, 0.079)
Male				
18-24 years	0.368	(0.294, 0.443)	0.438	(0.361, 0.515)
25-34 years	0.386	(0.325, 0.446)	0.439	(0.379, 0.499)
35-44 years	0.302	(0.246, 0.359)	0.340	(0.282, 0.397)
45-45 years	0.225	(0.172, 0.277)	0.267	(0.211, 0.322)
55-64 years	0.134	(0.085, 0.183)	0.174	(0.125, 0.222)
≥65 years	0.081	(0.046, 0.116)	0.071	(0.038, 0.104)

Table S3. Weighted frequency distributions of the potential risk and protective COVID-19 related factors for suicide ideation indication by measurement wave.

	Wave 1		Wave 2	
Suicide ideation:	unindicated n (%)	indicated n (%)	unindicated n (%)	indicated n (%)
Household composition				
Alone	1,184.2 (79.8)	299.8 (20.2)	1,179.9 (77.3)	346.3 (22.7)
With children	1,894.2 (70.5)	792.0 (29.5)	1,685.6 (65.1)	903.6 (34.9)
With others	3,594.4 (77.6)	1,039.3 (22.4)	3,673.8 (74.8)	1,235.8 (25.2)
Essential worker				
No	5,158.2 (79.2)	1,353.9 (20.8)	4,921.7 (76.4)	1,524.1 (23.6)
Yes: health	508.7 (62.7)	302.6 (37.3)	477.7 (59.8)	321.5 (40.2)
Yes: other	876.4 (67.3)	426.2 (32.7)	1,019.6 (63.7)	580.4 (36.3)
Self-isolation/quarantine				
No	2,529.6 (79.5)	652.8 (20.5)	3,625.4 (78.7)	978.5 (21.3)
Yes: case/symptoms-free	3,495.8 (79.7)	891.3 (20.3)	2,220.2 (73.3)	807.2 (26.7)
Yes: case or symptoms	495.3 (52.2)	454.1 (47.8)	593.3 (50.5)	582.4 (49.5)
Financial losses				
No	3,034.1 (81.1)	708.9 (18.9)	3,824.5 (80.0)	958.2 (20.0)
Yes	3,257.6 (72.8)	1,214.3 (27.2)	2,519.5 (65.0)	1,355.2 (35.0)
Unsure/unknown	383.2 (64.8)	208.0 (35.2)	197.3 (53.4)	172.3 (46.6)
Threat perceived for oneself and/or family				
High	2,031.5 (68.5)	936.0 (31.5)	2,330.0 (68.1)	1,089.7 (31.9)
Otherwise	4,456.9 (80.0)	1,112.6 (20.0)	4,035.7 (75.7)	1,298.6 (24.3)
Threat perceived for country and/or world				
High	4,711.7 (75.7)	1,511.5 (24.3)	4,853.9 (74.1)	1,698.7 (25.9)
Otherwise	1,740.7 (76.2)	544.1 (23.8)	1,498.4 (68.8)	681.0 (31.2)
Being a victim of stigma				
No	5,384.5 (81.5)	1,222.7 (18.5)	5,854.5 (78.1)	1,643.1 (21.9)
Yes	626.0 (49.9)	628.7 (50.1)	449.8 (40.6)	657.6 (59.4)
Decline to answer	664.4 (70.4)	279.7 (29.6)	237.0 (56.1)	185.1 (43.9)
Level of information about COVID-19				
High	2,139.2 (76.8)	645.3 (23.2)	2,032.0 (72.7)	761.2 (27.3)
Otherwise	4,535.7 (75.3)	1,485.8 (24.7)	4,509.3 (72.3)	1,724.5 (27.7)
Trust in authorities score				
Q1 (low)	1,662.9 (70.4)	698.9 (29.6)	1,588.5 (67.5)	766.2 (32.5)
Q2	1,597.6 (75.3)	524.3 (24.7)	1,403.4 (70.8)	578.1 (29.2)
Q3	1,716.7 (79.2)	449.6 (20.8)	1,690.7 (74.9)	565.9 (25.1)
Q4 (high)	1,697.7 (78.7)	458.3 (21.3)	1,858.6 (76.4)	575.7 (23.6)
Internet-based social media as a regular source of information				
Often/always	1,790.2 (66.7)	894.9 (33.3)	1,601.6 (60.4)	1,050.3 (39.6)
Sometimes/never	4,623.0 (80.0)	1,157.9 (20.0)	4,656.4 (77.7)	1,333.6 (22.3)
Friends/family/co-workers as a regular source of information				
Often/always	2,516.0 (71.6)	998.5 (28.4)	2,586.2 (68.9)	1,165.9 (31.1)
Sometimes/never	4,039.6 (79.0)	1,073.9 (21.0)	3,842.5 (75.5)	1,248.4 (24.5)
Sense of coherence				
Stronger (5-6)	2,437.5 (92.0)	212.2 (8.0)	2,488.2 (91.5)	231.0 (8.5)
Weaker (0-4)	4,237.4 (68.8)	1,918.9 (31.2)	4,053.0 (64.3)	2,254.7 (35.7)
